# *Lactobacillus casei* SYF-08 Protects Against Pb-Induced Injury in Young Mice by Regulating Bile Acid Metabolism and Increasing Pb Excretion

**DOI:** 10.3389/fnut.2022.914323

**Published:** 2022-06-28

**Authors:** Zhenhui Chen, Ziyu Tang, Jingjing Kong, Lixuan Chen, Jiaxin Liu, Yunting Li, Wanwen Huang, Wendan Li, Junlin Wu, Wei Zhao, Xiaojing Meng, Hongying Fan

**Affiliations:** ^1^Department of Microbiology, Guangdong Provincial Key Laboratory of Tropical Disease Research, School of Public Health, Southern Medical University, Guangzhou, China; ^2^Department of Occupational Health and Occupational Medicine, Guangdong Provincial Key Laboratory of Tropical Disease Research, School of Public Health, Southern Medical University, Guangzhou, China; ^3^Guangdong Huankai Microbial Science and Technology Co., Ltd., Guangzhou, China; ^4^BSL-3 Laboratory, Guangdong Provincial Key Laboratory of Tropical Disease Research, School of Public Health, Southern Medical University, Guangzhou, China

**Keywords:** Pb poisoning, *Lactobacillus casei* SYF-08, brain damage, intestinal microflora, bile acid metabolism, FXR, intestinal inflammation

## Abstract

Pb poisoning affects infant growth and development. However, dimercaptosuccinic acid (DMSA) as the current therapy for Pb poisoning exerts relatively significant toxic side effects in infants. Therefore, identifying a non-toxic treatment in this regard is particularly important. In this study, we aimed to investigate the therapeutic effect of an infant feces-derived probiotic strain, *Lactobacillus casei* SYF-08 (SYF-08), on Pb poisoning in young mice. The Pb levels in the organisms were detected *via* inductively coupled plasma mass spectrometry, while the therapeutic effect of SYF-08 on Pb-induced neural system damage was explored *via* the Morris water maze test, hematoxylin-eosin staining, and immunohistochemistry. Additionally, the molecular mechanisms underlying the protective effects of SYF-08 against Pb-induced intestinal damage were also explored *via* histological staining, 16S rRNA sequencing, untargeted metabolomics, qRT-PCR, and western blotting. *In vivo* experiments revealed that SYF-08 reduced blood and bone Pb levels and increased urinary Pb excretion. Additionally, SYF-08 alleviated Pb-induced pathological damage to the brain and ultimately improved the learning and cognitive abilities of the young mice. This treatment also restored intestinal microflora dysbiosis, regulated bile acid metabolism, and inhibited the FXR-NLRP3 signaling pathway. It also resulted in fewer adverse events than the DMSA treatment. In conclusion, our results provided valuable insights into the therapeutic role of SYF-08 in Pb poisoning and also suggested that its administration can significantly alleviate the Pb-induced damage.

## Introduction

Pb, which is a non-essential and naturally occurring heavy metal with cumulative toxicity, can stably exist in the soil, air, and water (1), and after entering the body through the digestive or respiratory tract, a small portion of it can be excreted through urine and feces, while the major portion circulates through the blood to other target organs (2). Therefore, Pb can affect the skeletal, urinary, neural, and digestive systems. Approximately 90–95% of Pb in the human body is stored in the bone tissue as Pb salts or Pb-protein complexes (3). Along with the bone remodeling process, bone Pb is continuously released from the bone, causing bone cell damage and apoptosis, and eventually leading to bone loss-related diseases, such as osteoporosis and fractures (4). Given that the urinary tract is the central site for Pb excretion, the kidney is also a target organ for Pb accumulation. Specifically, Pb can cause chronic kidney disease and also reduce glomeruli number and volume (5). Additionally, Pb poisoning has also been associated with neurological disorders. For example, it can cause aggressive behavior, anxiety, and depression, and affect learning and cognition (6). It has also been reported that the intestine is a vital organ for Pb absorption, and that Pb can cause significant intestinal damage (7).

Reversing or eliminating the harmful effects of Pb once it enters the target organ is challenging. Therefore, the prevention of Pb poisoning is more significant and beneficial than its treatment. The existing treatment methods for Pb poisoning include the use of various chelating agents and antioxidants. In this regard, dimercaptosuccinic acid (DMSA) is the most effective and safe chelator of Pb. It has a strong affinity for heavy metals, a reasonable lipid-water distribution coefficient that enables it to cross cell membranes, and good water solubility. Its use is also easy, and it is characterized low metabolism (8). However, 60% of patients on DMSA experience temporary and moderate increases in transaminase activity, and 6% experience a skin reaction. It has also been observed that DMSA increases urinary copper and zinc excretion (9). Therefore, the identification of new treatment methods with minor side effects for the management of Pb poisoning is necessary.

Lactic acid bacteria (LAB) constitute a group of gram-positive bacteria that are phylogenetically located in the Clostridia branch, with <50% GC content in their DNA (10). LAB are not only present in foods, but can also be found in the environment as well as in the human gut (11). It has also been observed that multiple LAB strains, especially *Lactobacillus rhamnosus, Lactobacillus casei*, and *Lactobacillus gasseri*, which are considered as probiotics, have been extensively studied considering their health-promoting properties (12, 13). Reportedly, LAB strains have the ability to inhibit the multiplication of pathogenic bacteria, increase the efficacy of the gut barrier, exert immunomodulatory effects, and interact with and regulate the function of other organs, including the liver, kidney, and brain (11, 14). However, some LAB strains are closely related to disease, including lung cancer, decsyed teeth, and metabolic disease (15, 16).

Apart from DMSA, another promising treatment method for Pb poisoning is the regulation of the composition of intestinal microflora, which is the first line of defense against the toxic effects of Pb (17). In the regard, the use of LAB, which is one of the primary strategies for changing intestinal microflora composition, has attracted the attention of several researchers of recent. Specifically, LAB improve intestinal barrier function by reducing the absorption of Pb in the intestine (18), and some LAB bind to Pb *via* electrostatic interactions. It has been observed that metal ions bind to cell walls and extracellular polysaccharides *via* active or inactive cell adsorption, ion exchange, complexation, chelation, and microprecipitation, which do not depend on temperature, metabolic energy, and metabolic inhibitors, but on available cell surface functional groups, the concentration of metal ions, surface charge, the particular metal cation, and the ligand (19, 20). Reportedly, *Lactobacillus plantarum* and *Lactobacillus bulgaricus* increase Pb removal rates in animal models (21, 22). In our previous study, we isolated a strain of *Lactobacillus casei* SYF-08 from newborn feces (23). This strain grew normally under a pH of 3.0–4.0 and exhibited the highest tolerance to Pb, with a minimum inhibitory concentration value of 2,500 mg/L. Further, the Pb-removal efficiency of SYF-08 was 51.00 ± 6.81% at an initial Pb concentration of 250 mg/L. Thus, SYF-08 also exerts protective effects against Pb toxicity in both C57BL/6 dams and offspring *in vivo*. However, whether it exerts a similar in young mice remains unknown.

Bile acid metabolism is an essential part of metabolism in the gut (24). The key bile acid-activated receptor, farnesoid × receptor (FXR) regulates the transcription of the genes involved in bile acid synthesis, absorption, uptake, and transport, thereby maintaining bile acid homeostasis (25, 26). Further, a decrease in its expression level results in the disturbance of bile acid homeostasis (26). It has also been demonstrated that LAB strains play a crucial role in bile acid metabolism. Specifically, Lactobacillus convert conjugated bile acids to their unconjugated counterparts and are also involved in the reactivation and enterohepatic circulation of compounds that have been eliminated through bile (27). Further, it has been observed that *Lactobacillus plantarum* CCFM8661 significantly induces hepatic bile acid synthesis, enhances bile flow and biliary glutathione output, and increases fecal bile acid excretion in mice, which in turn increases biliary Pb output and enhances fecal Pb excretion (28). This regulation is associated with the FXR and fibroblast growth factor 15 (FGF15) signaling pathway. However, it is still unclear whether *Lactobacillus casei* SYF-08 protects against Pb poisoning through bile acid metabolism and the FXR signaling pathway. Therefore, in this study, we aimed to clarify the underlying mechanisms in this regard using young mice.

## Materials and Methods

### Bacterial Strain and Culture Condition

The isolation and characterization of *Lactobacillus casei* SYF-08, which is a healthy newborn-derived probiotic, were performed as previously reported (23). The strain was cultured in the de Man, Rogosa, and Sharpe (MRS; Huankai Microbial Sci. and Tech, Guangdong, China) medium at 37°C in a bacterial incubator (Thermo Fisher Scientific, Waltham, MA, USA).

### Animal Model and Acute Single Dose Oral Toxicity Test

C57BL/6 mice were purchased from the Southern Medical University Laboratory Animal Center, China, and raised under specific pathogen-free conditions with food and drinking water provided *ad libitum*. The experimental protocol was approved by the Institutional Animal Care and Use Committee of Southern Medical University (SMUL2018037) and all *in vivo* experiments were performed in accordance with the guidelines of our institution for the use of laboratory animals. Acute single dose oral toxicity tests were performed in accordance with OECD guidelines for testing chemicals (47). In brief, male C57BL/6 mice (3–4 weeks old, *n* = 10) were divided into two groups of five mice. Thereafter, mice in the SYF-08 treatment group were orally administered SYF-08 at 1 × 10^11^ CFU/200 μL/day *via* gavage, while those in the PBS control group were orally administered 200 μL saline per day. Thereafter, mortality, signs of toxicity, and body weight changes were monitored for 14 days. Further, to establish the Pb-poisoning animal model, 3-week-old male mice (*n* = 36) were randomly divided into six groups with no blinding. Thereafter, the mice were treated with Pb (100 ppm of lead acetate) *via* their drinking water. After the establishment of the model, the animals in the DMSA group were treated with DMSA (150 mg/kg/day; Aladdin, Shanghai, China) *via* gavage, while those in the SYF-08 group were treated with *Lactobacillus casei* SYF-08 (1 × 10^10^ CFU/100 μL/day) *via* gavage. At 4-day intervals during the experimental period, fecal and urine samples were collected for analysis and at the end of the treatments, the mice were euthanized using phenobarbital sodium, and their organs, blood, and bones were collected. In particular, the organs were weighed and embedded in paraffin for further analysis.

### Determination of Pb, Ca^2+^, Mg^2+^ Levels in Blood, Bone, Fecal, and Urine Samples

The tissue samples were digested in concentrated HNO_3_ and H_2_O_2_ (2:1) using a microwave digestion system (MARS; CEM, United Kingdom). Specifically, the samples were digested in a tube at 110°C for 6 h, after which the cover of the digestion tube was opened for 8 h at 100°C to remove the acid. After digestion, ultrapure water was used to maintain the volume at 5 mL. Finally, Pb levels were determined *via* inductively coupled plasma mass spectrometry (ICP-MS; Thermo Fisher Scientific), while Ca^2+^ and Mg^2+^ levels in blood were determined using the iCAP 7000 Series ICP-OES (Thermo Fisher Scientific).

### Morris Water Maze Test

Cognitive function was tested using the Morris water maze test (TSE Systems, Bad Homburg, Germany) (29). The maze consisted of a black plastic circular tank (120-cm diameter) containing a hidden platform with a diameter of 10 cm placed in the target quadrant and submerged 1 cm below the water surface for all the tests. The mice received a 1-day adaptability trial before the hidden platform trial was performed, during which the mice were placed in every quadrant to find the hidden platform for 60 s. Thereafter, they were allowed to swim until the hidden platform was found within 60 s for 4 days. Further, in the probe trial, the platform was removed and the mice were placed in the tank and the number of platform crossings, and the time and percentage of time and path in the target quadrant were recorded for 120 s.

### Hematoxylin-Eosin Staining and Immunohistochemistry

Hematoxylin-eosin (HE) staining was performed according to the manufacturer's instructions. Further, immunohistochemistry (IHC) staining was performed to determine protein expression levels using the corresponding antibodies, namely, rabbit anti-NeuN polyclonal antibody (26975-1-AP, 1:8,000, Proteintech, Wuhan, China) and rabbit anti-FXR polyclonal antibody (25055-1-AP, Proteintech, Wuhan, China). Further, sections stained for IHC were examined using the DAB staining system (Maxim, Fuzhou, China). The staining intensity was evaluated using ImageJ software version 1.8.0 (National Institutes of Health, Bethesda, MD, USA) (30).

### 16S rRNA Sequencing of Intestinal Contents

Total genomic DNA was extracted from the intestinal contents as previously described (31). Briefly, 200 mg of intestinal content was used for DNA extraction according to the manual accompanying the TIANamp Stool DNA Kit (Tiangen, Beijing, China). The V3–V4 region of the 16S rRNA gene was amplified using 338F and 806R primers and the samples were sequenced using an Illumina HiSeq 2500 platform (Illumina, San Diego, CA, USA) by MAGIGENE (Guangzhou, China). Paired-end reads were merged *via* the fast length adjustment of short reads, and sequence analysis was performed using UPARSE (32). Thereafter, sequences with ≥97% similarity were assigned to the same operational taxonomic units (OTUs) (32, 33). Further, the QIIME software was used to select the representative sequences from each OTU, which thereafter, were subjected to comparison and annotation based on the SILVA database (v138) (34). The alpha diversity index of the bacterial communities was determined using usearch-alpha_div (v10) in software USEARCH (35), while the beta diversity index was determined using R package vegan (v1.17) and the unweighted pair-group method with arithmetic mean (36). Further, linear discriminant analysis effect size (LEfSe) was performed using LEfSe software (v1.0) (37).

### Untargeted Metabolomics

Approximately 100 μL of serum was placed in an Eppendorf (EP) tube followed by the addition of 400 μL of a methanol solution (Sigma-Aldrich, St. Louis, MO, USA) with an 80% volume fraction. Next, the tube was scrolled and shaken, and thereafter, placed on ice for 5 min. This was followed by centrifugation at 15,000 ×g and 4°C for 20 min, after which the supernatant was collected and added to ultrapure MS water, and the methanol content was diluted to 53%. Centrifugation was again performed at 4°C and 15,000 ×g for 20 min. Thereafter, the supernatant was collected and analyzed *via* liquid chromatography-mass spectrometry (LC-MS; Agilent Technologies, Santa Clara, CA, USA) using a Hypesil GOLD column (C18) at a flow rate of 0.2 mL/min and a temperature of 40°C. In the positive mode, mobile phase A was 0.1% formic acid, while mobile phase B was methanol and in the negative mode, mobile phase A was 5 mM ammonium acetate (pH 9.0), while mobile phase B was methanol. The conditional scanning range of the MS, i.e., the mass charge ratio (m/z) range, was 100–1,500. The process of metabolite identification normalization was conducted by Novogene (Beijing, China) with mzCloud, mzVault, and MassList databases as the reference libraries.

### RNA Isolation and qRT-PCR

Total RNA was extracted from colon segments (0.1 g) using TRIzol reagent (Takara Bio, Tokyo, Japan). Thereafter, the extracted RNA was converted to cDNA using a reverse transcription kit (Takara Bio) and gene expression was then determined using the qPCR SYBR Green Master Mix system (Takara Bio) and the 7500 real-time quantitative PCR system (Applied Biosystems, Thermo Fisher Scientific). GAPDH was used for normalization and the relative quantification of the expression levels of the target genes were performed using the 2^−ΔΔCT^ method (38). The primers used are listed in Table 1.

**Table 1:** Primers used for the real-time PCR.

**Gene symbol**	**Forward primer**	**Reverse primer**
GAPDH	AGGTCGGTGTGAACGGATTTG	GGGGTCGTTGATGGCAACA
FXR	GGCAGAATCTGGATTTGGAATCG	GCCCAGGTTGGAATAGTAAGACG
FGF15	ATGGCGAGAAAGTGGAACGG	GGACCAGCGGAGTACAGGT
NLRP3	ATTACCCGCCCGAGAAAGG	TCGCAGCAAAGATCCACACAG
IL-1β	GCAACTGTTCCTGAACTCAACT	ATCTTTTGGGGTCCGTCAACT

### Protein Extraction and Western Blotting

Proteins were extracted from 0.5-g samples of ileum tissue using a protein extraction kit (Beyotime Biotechnology, China) and subsequently used for western blot analysis, which was performed in accordance with manufacturer's instructions. The dilution ratio of all the primary antibodies, including rabbit anti-NLPP3 polyclonal antibody (19771-1-AP, Proteintech), rabbi anti-Caspase 1 polyclonal antibody (22915-1-AP, Proteintech), rabbit anti-IL-1β polyclonal antibody (16806-1-AP, Proteintech), and rabbi anti-FGF15 polyclonal antibody (ab229630, Abcam, Cambridge, MA, USA) and mouse anti-β-actin polyclonal antibody (CST4970; CST, Danvers, MA, USA), was 1:1,000, while the dilution ratio of all the secondary antibodies, anti-rabbit IgG, HRP-linked antibody (CST7074, CST) was 1:2,000. Enhanced chemiluminescence was used as detection chemistry.

### Statistical Analysis

All data are expressed as mean ± standard deviation, and statistical significance was set at *P* ≤ 0.05. Statistical differences between the experimental groups were analyzed using Student's *t*-test, and all statistical analyses were performed using SPSS (version 22.0; IBM, NY, USA). All experiments were performed in triplicate.

## Results

### *Lactobacillus casei* SYF-08 Increases Pb Excretion and Reduces Pb Accumulation

The acute single dose oral toxicity test indicated that *Lactobacillus casei* SYF-08 (SYF-08), a strain isolated from newborn feces, was not toxic to the host. After seven days of daily SYF-08 administration *via* gavage, blood and major organs were harvested for further analysis. The results indicated that SYF-08 had no adverse effects on the hematological factors and serum biochemical factors of the mice (Supplementary Figure 1A). Further, there were no obvious pathological changes in the organs from the SYF-08-treated mice relative to those from the PBS control mice. Furthermore, no significant differences in organ coefficients were observed (Supplementary Figures 1B,C). The treated mice showed overall 103–109% body weight gain during the observation period, and there was no statistically significant difference between the SYF-08-treated and PBS group mice in this regard (Supplementary Figure 1D). Thus, SYF-08 administration did not induce death during the study period (Supplementary Figure 1E).

**Figure 1:**
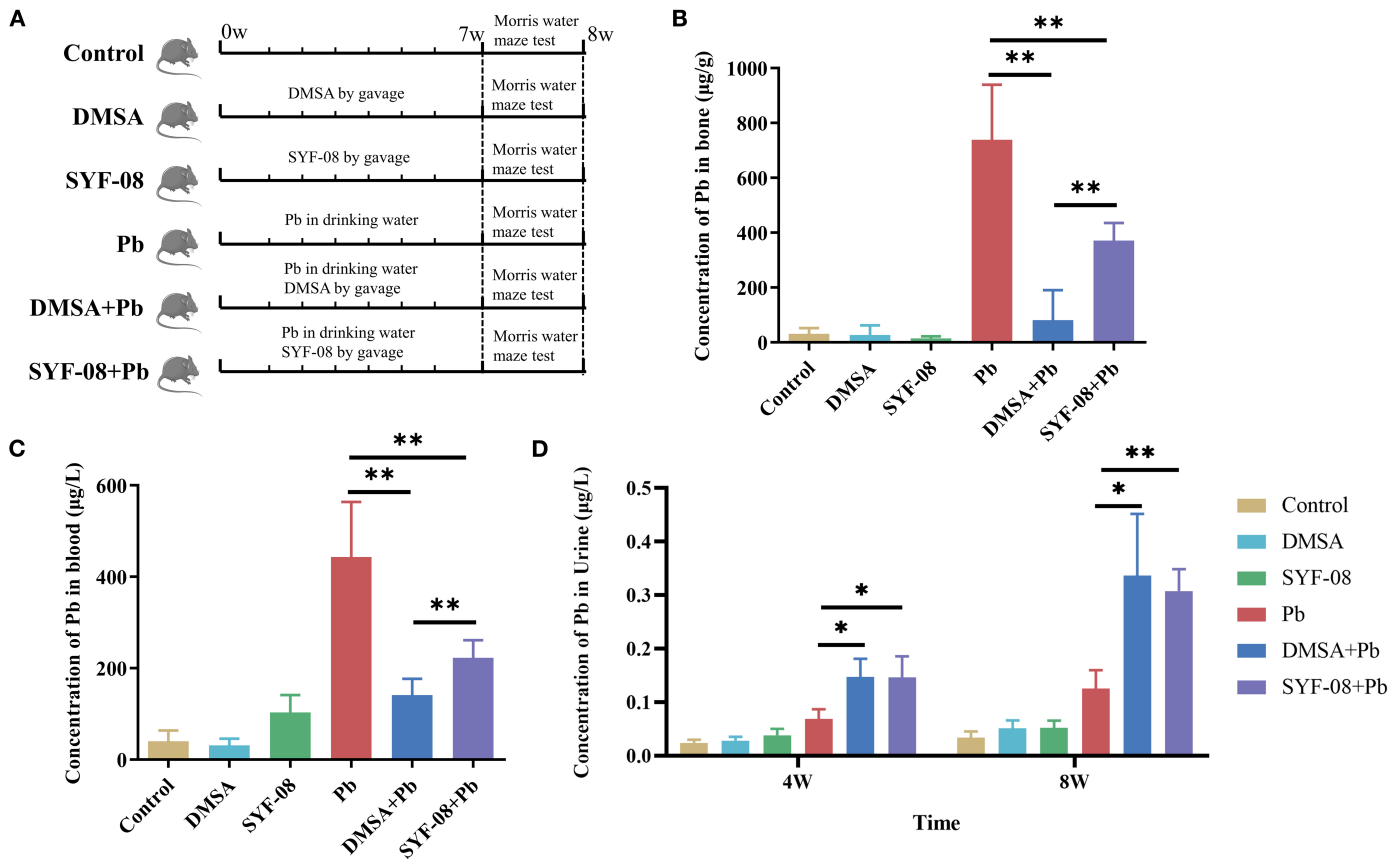
Animal model and Pb levels in organisms. **(A)** Overview of the study design and samples collection. The concentration of Pb in **(B)** bone, **(C)** blood, and **(D)** urine. *P < 0.05; **P < 0.01.

Previously, we also found that SYF-08 is a promising probiotic candidate against Pb toxicity in both C57BL/6 dams and offspring *in vivo* (23). However, it remains unknown whether it exerts a similar probiotic effect in young mice. Therefore, we established a Pb poisoning model in young mice and treated the animals with SYF-08 or DMSA (Figure 1A). The concentration of Pb in the bone, blood, and urine of mice in the Pb treatment group indicated the successful establishment of the Pb poisoning model (Figures 1B–D). Further, compared with the Pb group, the concentrations of Pb in the bone and blood of mice in the DMSA + Pb and SYF-08 + Pb groups were significantly reduced (Figures 1B,C). Further, the therapeutic effect of DMSA treatment significantly higher than that of SYF-08 (Figures 1B,C). Furthermore, the urine Pb levels corresponding to mice in the DMSA + Pb and SYF-08 + Pb groups were significantly higher than that corresponding to mice in the Pb group at weeks 4 and 8 (Figure 1D). Generally, we also observed that SYF-08 enhanced urine Pb level and reduced Pb accumulation in the bone and blood of the treated mice. Our results also indicated significantly reduced Ca^2+^ and Mg^2+^ levels in the blood of mice in the DMSA group, indicating that Ca^2+^ and Mg^2+^ loss was a side effect of the DMSA treatment (Supplementary Figure 2). The calculation of the weight gain on the last day before euthanization showed that the least weight gain corresponded to mice in the Pb group (Supplementary Figure 3). Thus, treatment with DMSA and SYF-08 reversed Pb-induced weight loss (Supplementary Figure 3).

### *Lactobacillus casei* SYF-08 Alleviates Damage in the Nervous System

**Figure 2:**
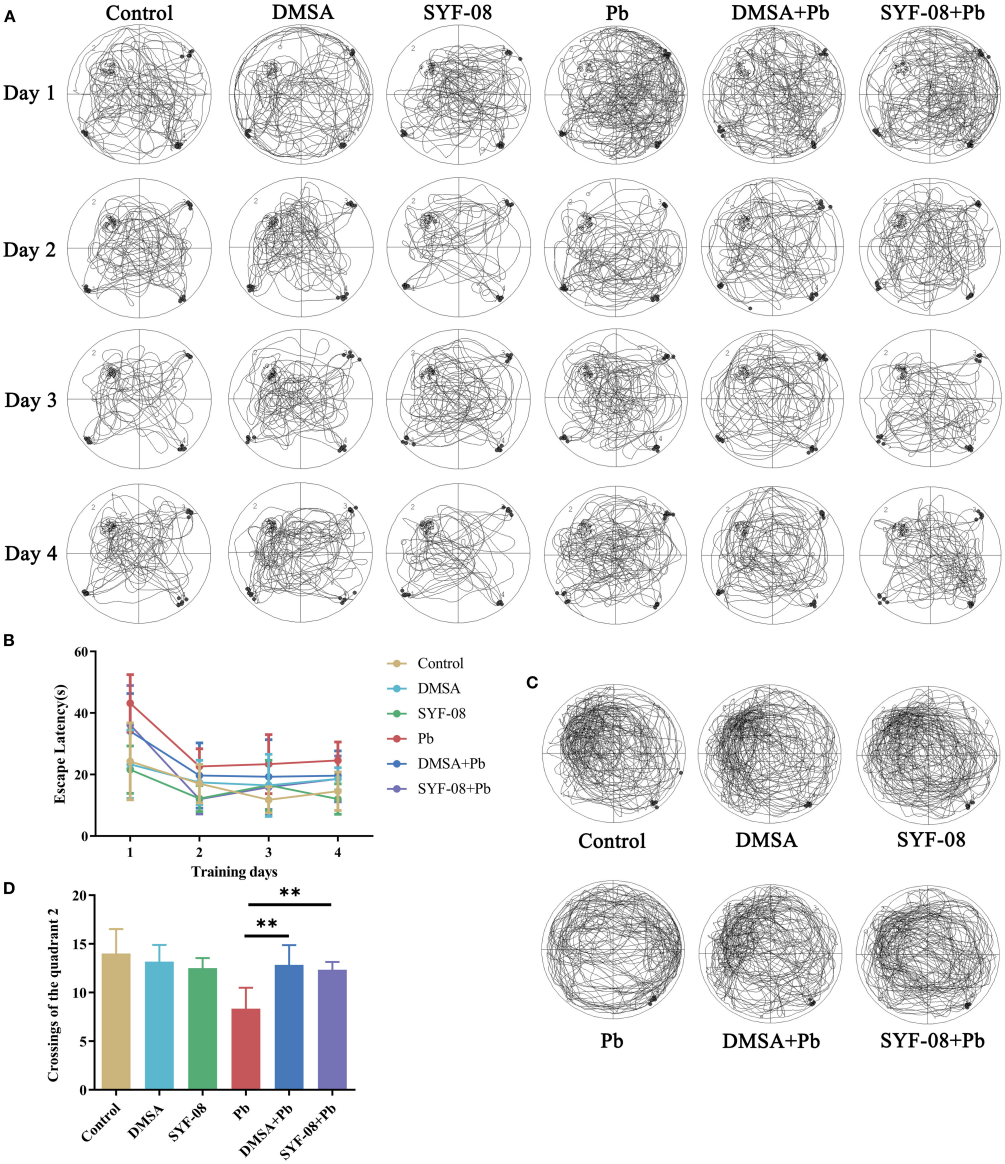
*Lactobacillus casei* SYF-08 protects against Pb-induced cognitive dysfunction. **(A)** Representative crossings of the test during the hidden platform trial. **(B)** The escape latency in the different groups from days 1 to 4. **(C)** Representative crossings of the test during the probe trial. **(D)** The number of crossings of the quadrant 2 in the different group. **P < 0.01.

Given that the brain is one of the main targets of Pb, we assessed the cognitive function of mice in each group using the Morris water maze test. The crossings of all the quadrants in the hidden platform training phase are shown in Figure 2A. The time to find the platform gradually decreased with an increase in the number of training days in each mice group (Figure 2B). Further, the crossings of all the quadrants in the hidden platform test phase are shown in Figure 2C, from which it is evident that, consistent with the training phase results, the quadrant 2 crossings corresponding to the Pb group were lower than those corresponding to the Control group, but increased after treatment with DMSA or SYF-08 (Figure 2D).

**Figure 3:**
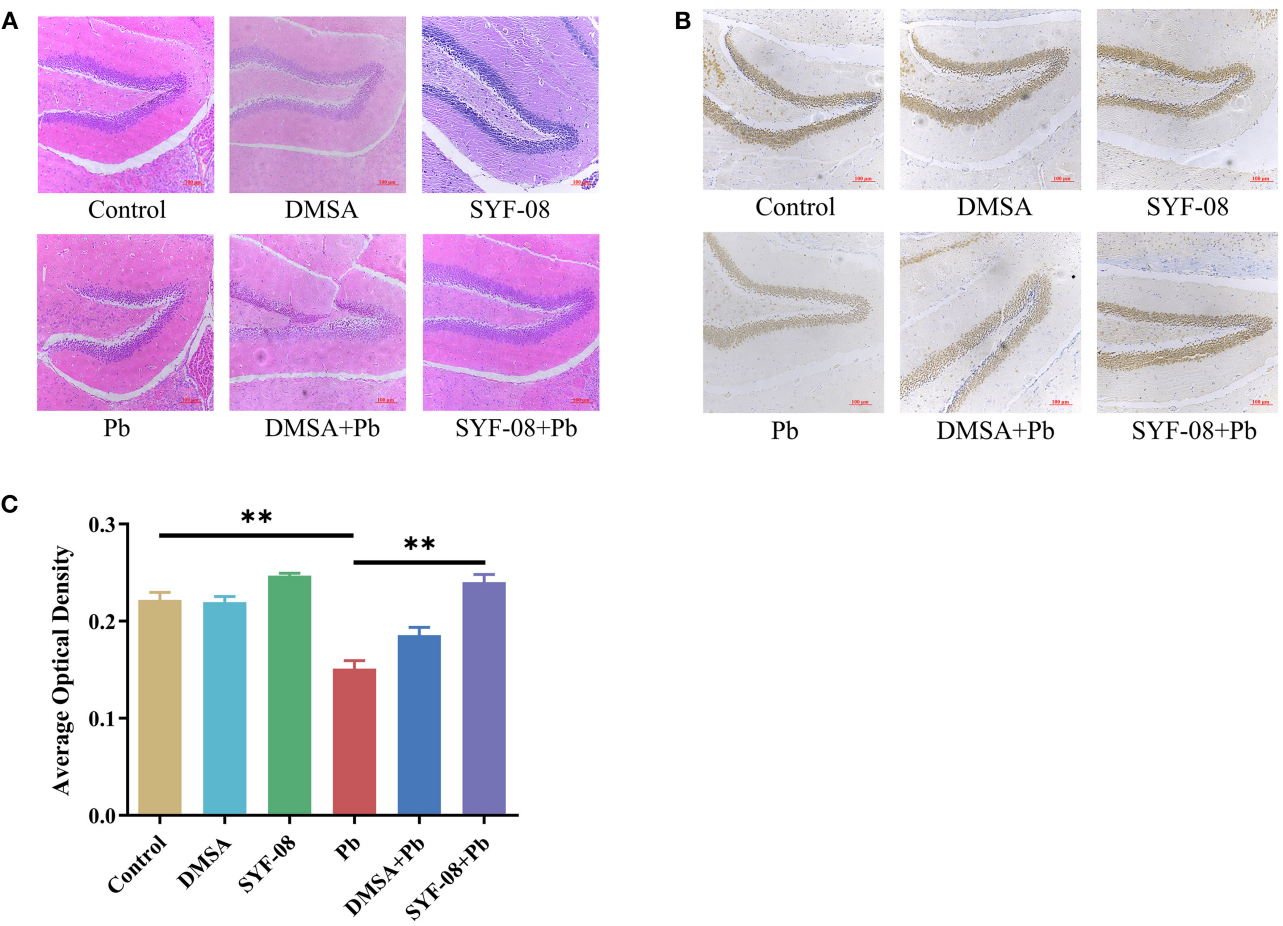
*Lactobacillus casei* SYF-08 protects against Pb-induced brain injury. **(A)** Representative HE staining of brain in the different groups (200×). **(B)** Representative IHC staining of NeuN-positive cells in the different groups (200×). **(C)** Avergae optical density of IHC staining. **P < 0.01.

Next, we used HE staining and IHC to detect pathological changes in the brain and explored whether SYF-08 alleviated Pb-induced nervous system damage (Figure 3). The results thus obtained indicated that brain tissues from mice in the Control, DMSA, and SYF-08 groups were characterized by a regular arrangement of neurons, regular morphology of glial cells and hippocampus, and showed the presence of a granular cell layer of the dental gyrus (Figure 3A). Conversely, mice in the Pb group showed severe pathological damage and atrophy as well as tissue cavitation in the hippocampus. Thus, these histopathological changes were reversed by the SYF-08 treatment. Although the DMSA + Pb treatment group also showed the attenuation of these histopathological changes, the outcomes were still worse and a more disordered structure was observed in the granular cell layer of the dental gyrus of mice in this group. Further, the DMSA + Pb group showed a higher degree of tissue cavitation than the SYF-08 + Pb group.

In this study, brain tissue samples were also subjected to IHC staining of NeuN, after which average optical densities were calculated. The results obtained are shown in Figures 3B,C, from which it is evident that the Pb and DMSA + Pb groups had a lower number of NeuN-positive cells than the Control group. However, the number of NeuN-positive cells corresponding to the SYF-08 + Pb group was significantly higher than that corresponding to the Pb group and similar to that corresponding to the Control group. Interestingly, the number of NeuN-positive cells corresponding to the DMSA + Pb group was lower than that corresponding to the SYF-08 + Pb group.

Thus, SYF-08 alleviated Pb poisoning-induced nervous system damage without causing any additional damage to nerve cells in the hippocampus, unlike DMSA.

### *Lactobacillus casei* SYF-08 Reduces Intestinal Damage and Restores Intestinal Microflora Dysbiosis

**Figure 4:**
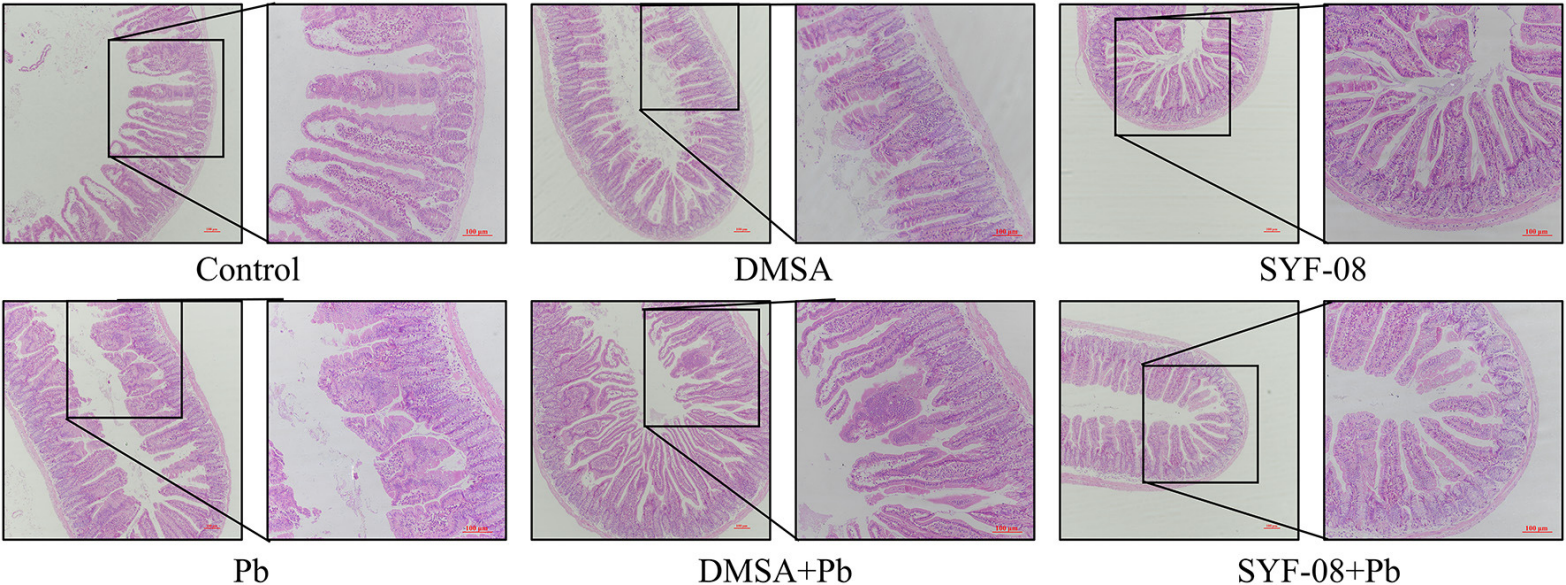
*Lactobacillus casei* SYF-08 protects against Pb-induced intestinal injury (100× and 200×).

The gut is an organ that has been increasingly recognized as one that plays an important role in minimizing the effects of Pb poisoning. In this study, we used HE staining to determine whether SYF-08 reduced intestinal damage. As shown in Figure 4, there were noticeable pathological changes in the small intestine, including morphological destruction or disappearance of intestinal villi, the fusion of adjacent intestinal villi, and atrophy or disappearance of the small intestinal gland and the central chylous duct. However, in the SYF-08 + Pb group, the mice recovered sufficiently from the pathological damage to the small intestine. This treatment also resulted in the restoration of the villus structure of the small intestine; however, a certain degree of disorder still existed. Further, the small intestinal epithelial cells appeared closely arranged, and the morphologies of the small intestinal gland and central chylous duct were normal. Regarding the DMSA + Pb group, the observed pathological injury to the intestine was more severe than that corresponding to mice the SYF-08 + Pb group. Further, the DMSA + Pb treatment resulted in shorter or fused intestinal villi, as well as the disappearance of the central chylous duct.

**Figure 5:**
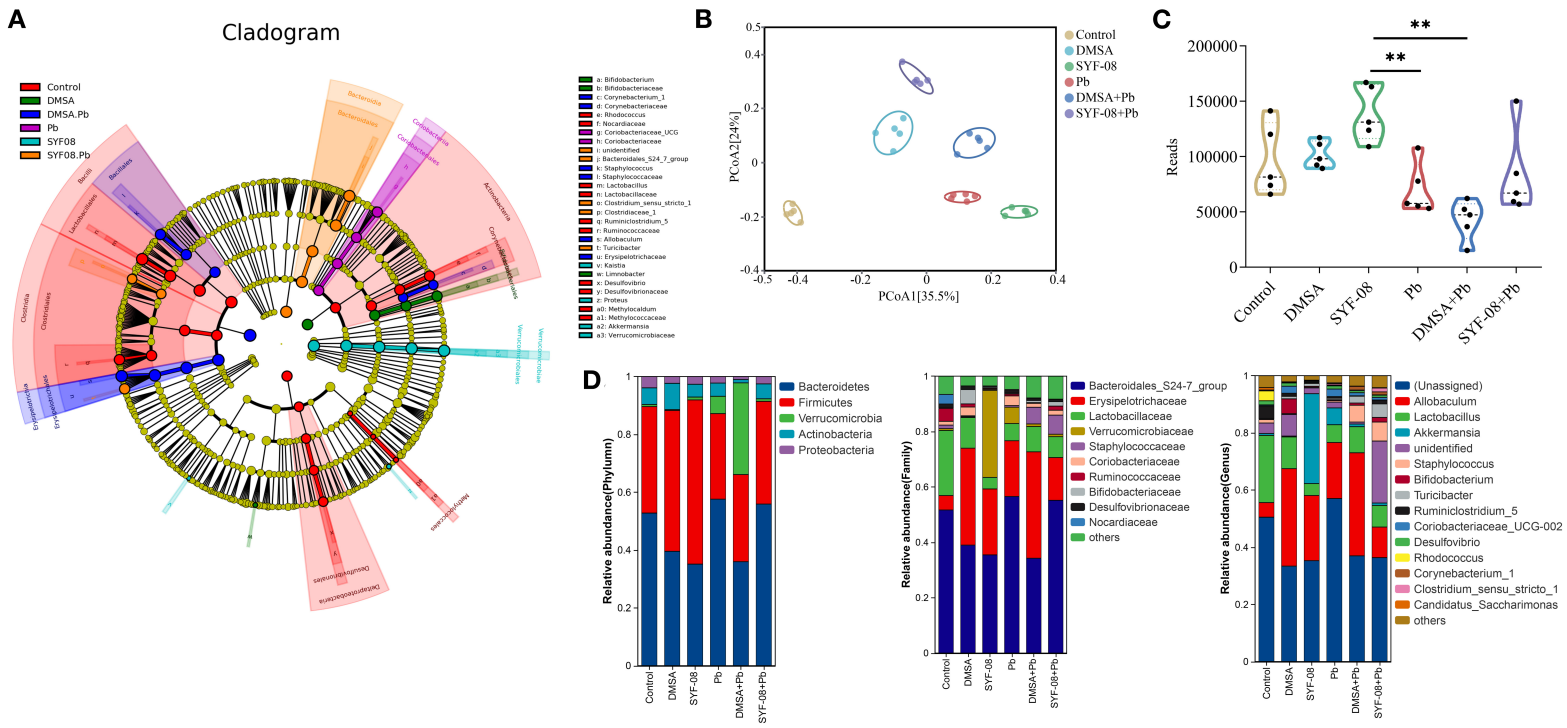
*Lactobacillus casei* SYF-08 restores Pb-induced intestinal microflora dysbiosis. **(A)** LEfSe analysis in the Control, Pb, SYF-08, DMSA, SYF-08 + Pb, and DMSA + Pb groups. **(B)** PCoA scatterplot displaying overall bacterial community composition in the different groups. **(C)** Reads indices in the different groups. **(D)** Relative abundance of intestinal microflora composition at different level. **P < 0.01.

To further investigate whether Pb affected the intestinal microflora and whether SYF-08 attenuated this effect, we conducted 16S rRNA sequencing on intestinal content samples from each group. The LEfSe analysis showed that *Lactobacillus, Lactobaillaceae, Ruminococcaceae, Rhodococcus*, and *Nocardiaceae* were enriched in the Control group, *Coriobacteriiaceae UCG, and Coriobacteriaceae* were significantly enriched in the Pb group, *Bacteroidales S24 7_group, Clostridiaceae*,, and *Turicibacter* were enriched in the SYF-08 + Pb group, and *Corynebacteriaceae, Staphylococcus, Erysipelotrichaceae*, and *Allobaculum* were enriched in the DMSA + Pb group (all |LDA score| > 4.0, Figure 5A). Further, PCoA analysis, with the Bray-Curtis index as the distance metric, revealed a cluster of intestinal microflora in the different groups (Figure 5B). Furthermore, the determination of α-diversity indices showed that the Reads indices corresponding to the Pb and DMSA + Pb groups were significantly lower than those corresponding to the SYF-08 group, indicating intestinal microflora dysbiosis in the Pb and DMSA + Pb groups, as well as its reversal following treatment with SYF-08 (Figure 5C). And the Chao1 indices corresponding to the DMSA + Pb and SYF-08 + Pb groups were significantly lower than those corresponding to the SYF-08 group (Supplementary Figure 4). Additionally, the relative abundance at phyla, family, and genus levels showed that the intestinal microflora composition of mice in the SYF-08 + Pb group was closer to that of mice in the Control group than to that of mice the Pb group (Figure 5D).

In summary, our results indicated that SYF-08 reduced intestinal damage and reversed intestinal microflora dysbiosis caused by Pb poisoning.

### Bile Acid Metabolism in the Blood is Closely Related to Pb Poisoning

**Figure 6:**
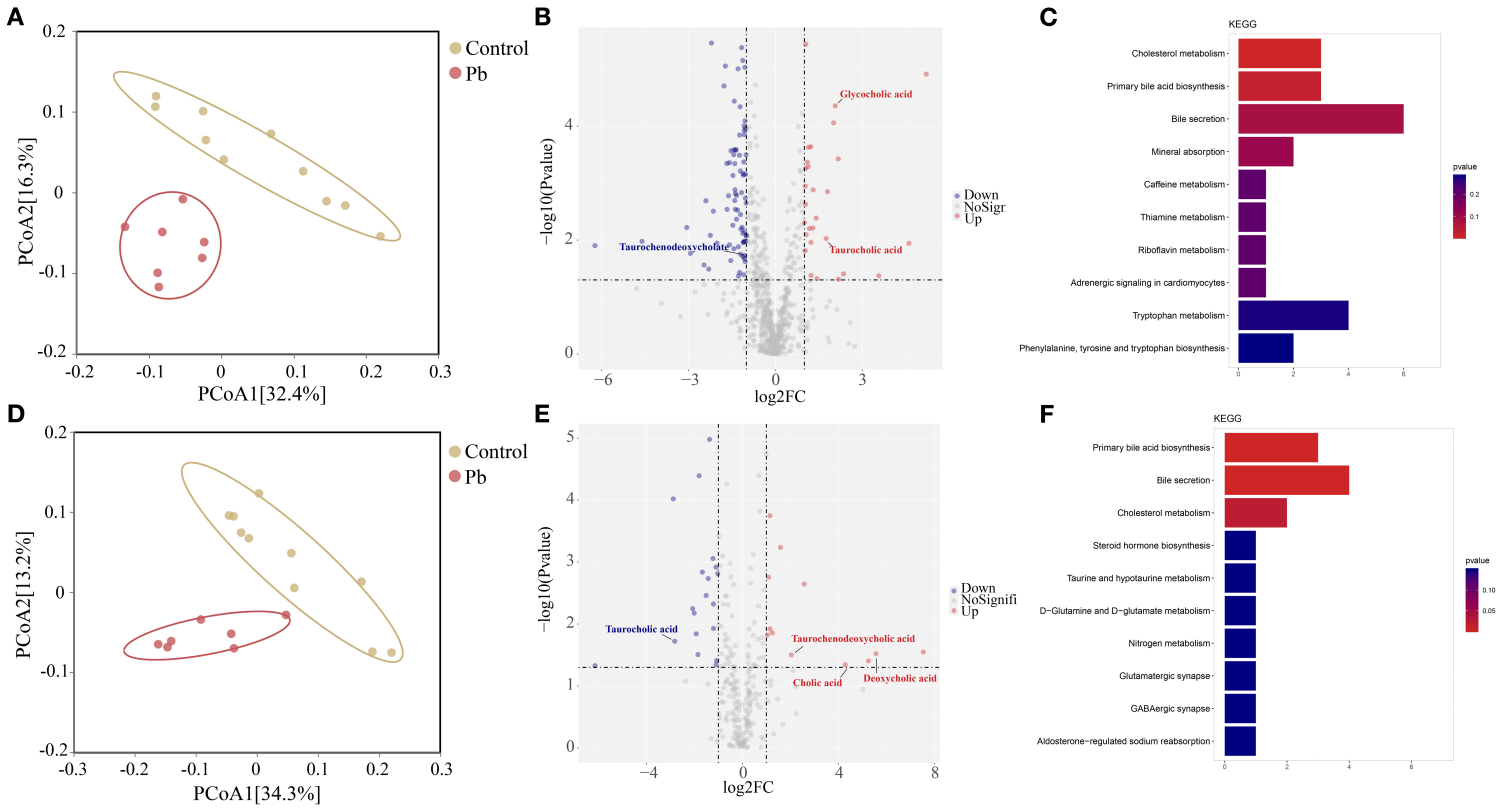
Bile acid metabolism is changed after Pb poisoning. **(A)** PCoA scatterplot displaying overall metabolites composition in postive mode. **(B)** Valcano plot of metabolites in postive mode. **(C)** Dot plot of enriched KEGG pathway in the positive mode. **(D)** PCoA scatterplot displaying overall metabolites composition in negative mode. **(E)** Valcano plot of metabolites in negative mode. **(F)** Dot plot of enriched KEGG pathway in the negative mode.

Blood bridges the nervous and digestive systems. In this study, we used untargeted metabolomics to explore changes in blood metabolites after Pb poisoning. Thus, PCoA analysis indicated a cluster of metabolites in the control and Pb groups (Figures 6A,D). Further, differential metabolites are shown in the volcano plot in Figures 6B,E. In the positive mode, glycocholic acid, taurocholic acid, and 26 other metabolites were upregulated, whereas taurochenodeoxycholate and 75 other metabolites were downregulated in the Pb group (Log_2_|FC| ≥ 1, *P* < 0.05, Figure 6B). Furthermore, in negative mode, taurochenodeoxycholic acid, deoxycholic acid, cholic acid, and nine other metabolites were upregulated, whereas 16 metabolites were downregulated in the Pb group (Log_2_|FC| ≥ 1, *P* < 0.05, Figure 6E). The KEGG pathway enrichment analysis of the differential metabolites in the positive and negative modes (*P* < 0.05) are shown in Figures 6C,F, respectively. From these figures, it was evident that bile acid metabolism-related pathways, including primary bile acid biosynthesis and bile secretion, showed the most significant *p*-values. Taken together, we found that Pb poisoning resulted in significant changes in bile acid metabolism-related pathways.

### *Lactobacillus casei* SYF-08 Downregulates the Pb-Activated FXR-NLRP3 Signaling Pathway

**Figure 7:**
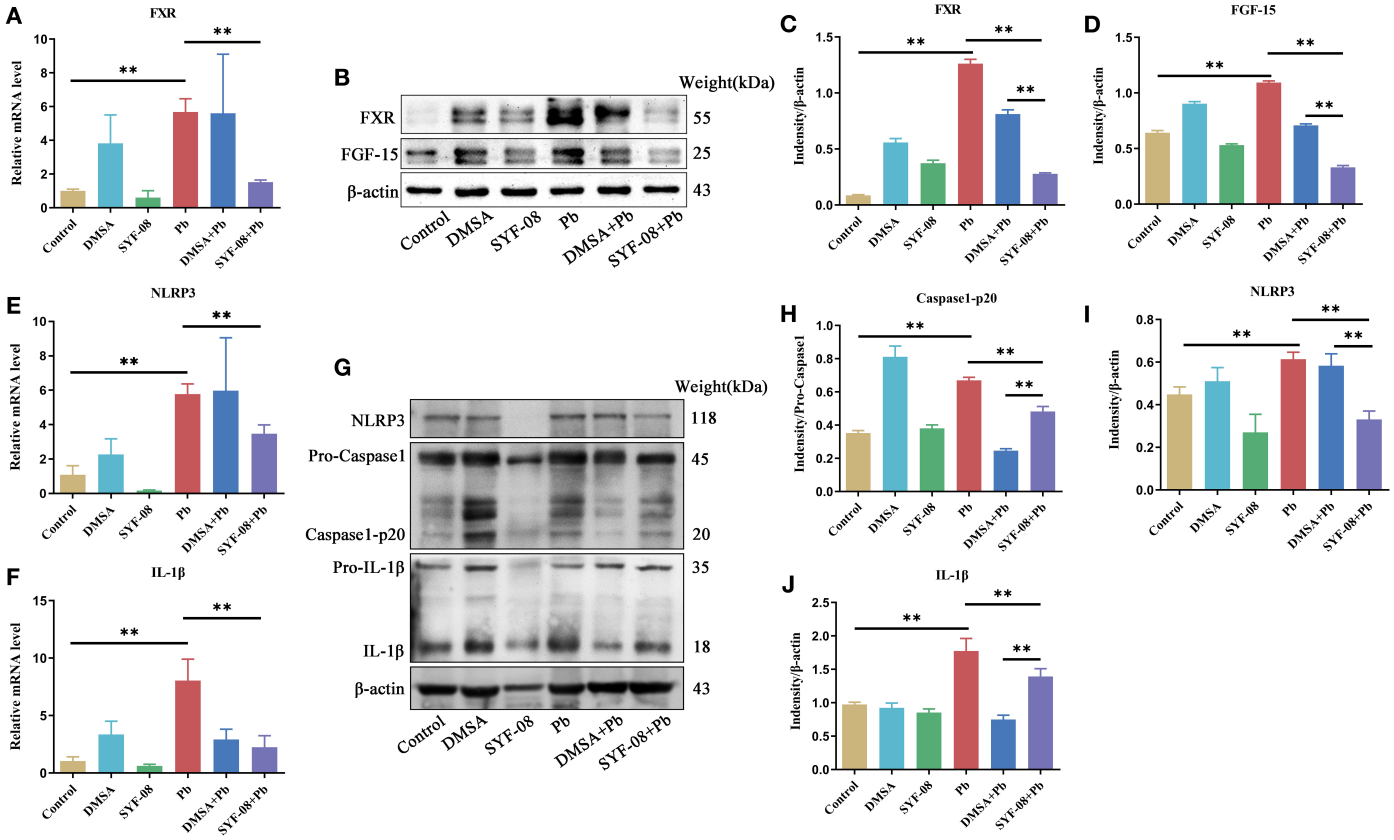
*Lactobacillus casei* SYF-08 inhibits the activated FXR-NLRP3 signaling pathway in the gut. **(A)** The transcript level of FXR in the different groups. **(B)** The protein level of FXR and FGF15 in the different groups. **(C)** Relative gray level of FXR. **(D)** Relative gray level of FGF-15. **(E)** The mRNA level of NLRP3 in different groups. **(F)** The mRNA level of IL-1β in different groups. **(G)** The protein level of NLRP3, Caspase 1 and IL-1β in the different groups. **(H)** Relative gray level of Caspase 1-p20. **(I)** Relative gray level of NLRP3. **(J)** Relative gray level of activated IL-1β. **P < 0.01.

Given that SYF-08 colonizes the gut, and bile acid metabolism occurs in the gut and liver, we investigated the FXR signaling pathway in the ileum and liver. Thus, we observed an increase in FXR mRNA level in the Pb group. However, SYF-08 treatment reversed this increase (Figure 7A). Consistent with the qRT-PCR results, FXR and FGF15 protein levels were upregulated in the Pb group, but were reversed after SYF-08 treatment (Figures 7B–D). Interestingly, the downregulation of the FXR signaling pathway was not observed in the DMSA + Pb group, indicating that SYF-08 played a unique role in this regard (Figures 7B–D). Additionally, NLRP3 inflammasome is downstream of the FXR signaling pathway. Thus, we explored changes in its mRNA level. The results obtained in this regard showed that the Pb group showed an increase in NLRP3 and IL-1β mRNA levels, which were reversed after SYF-08 treatment (Figures 7E,F). Further, the results of western blot analysis showed increased NLRP3, Caspase 1-p20, and IL-1β protein levels in mice in the Pb group; however, these changes were reversed in mice in the SYF-08 + Pb and DMSA + Pb groups (Figures 7G–J). Overall, these results indicated that SYF-08 downregulated the Pb-activated FXR-NLRP3 signaling pathway in the ileum.

Furthermore, we explored changes in the liver after SYF-08 and DMSA treatment. The liver organ indices corresponding to the different groups are shown in Supplementary Figure 5A. Specifically, the Pb group showed a decrease in liver weight, which was reversed in the SYF-08 + Pb group, indicating that SYF-08 exerted a protective effect on the liver (Supplementary Figure 5A). Further, HE staining of liver tissues (Supplementary Figure 5B) showed structural changes in the liver sinusoids as well as mild steatosis in the livers of mice in the DMSA and DMSA + Pb groups. IHC staining of FXR was also performed on liver tissues, and average optical densities were calculated (Supplementary Figures 5C,D). The results showed a higher level of FXR expression in the Pb group than the Control group. Additionally, the FXR expression level corresponding to the SYF-08 + Pb group was significantly lower than that corresponding to the Pb group, but similar to that corresponding to the Control group.

**Figure 8:**
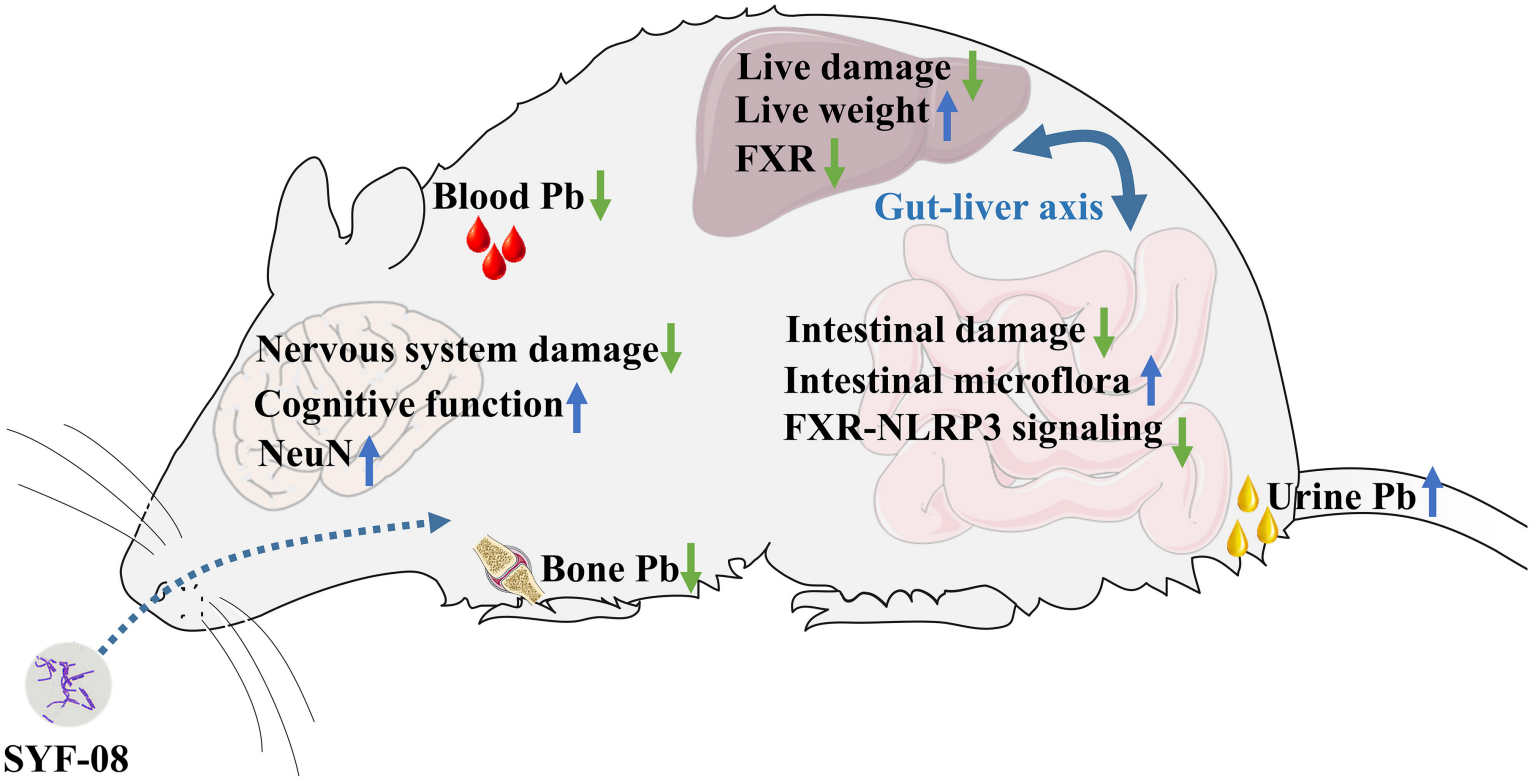
The summary of the research.

In summary, we observed that *Lactobacillus casei* SYF-08 reduced blood and bone Pb levels and increased urinary Pb excretion. It also alleviated pathological damage to the brain and ultimately improved the learning and cognitive abilities of young mice with Pb poisoning. Additionally, it alleviated pathological damage to the liver by inhibiting FXR expression and also restored dysbiosis of intestinal microflora, regulated bile acid metabolism, and inhibited the FXR-NLRP3 signaling pathway (Figure 8).

## Discussion

In this study, we used *Lactobacillus casei* SYF-08, previously isolated from neonatal feces, to treat Pb poisoning in young mice. Thus, we observed that it effectively enhanced Pb excretion, reduced Pb accumulation, improved cognitive behavior, recovered brain and intestinal damage, restored intestinal microflora dysbiosis, and regulated bile acid metabolism by downregulating the FXR-NLRP3 signaling pathway. Further, compared with the DMSA treatment, the SYF-08 treatment resulted in fewer adverse effects on the brain and liver, including disordered structure in the granular cell layer of the dental gyrus and structural changes in the liver sinusoids, as well as better therapeutic efficacy. Thus, this study established SYF-08 as an effective and safe therapeutic strategy for protecting adolescents from Pb poisoning.

The detection of Pb levels in the bone, blood, and urine of the experimental animals indicated that the SYF-08 treatment enhanced Pb excretion *via* urine and reduced Pb accumulation in the bone and blood. Reportedly, probiotics, including *Bacillus subtilis, Lactobacillus plantarum* LP33, *Lactobacillus bulgaricus, Lactobacillus plantarum* CCFM8661, and LAB strain-96 confer significant protective effects against Pb toxicity by preventing the bioaccumulation of Pb and promoting its excretion (22, 39–41). This suggests that the protective effect of *Lactobacillus* in Pb poisoning is worth investigating, and that its therapeutic effect in this regard may be the combined effect of its structure and function.

Additionally, the detection of brain damage in the Pb-poisoning mouse model showed that SYF-08 alleviated this damage in the brain, with no additional damage to the nerve cells in the hippocampus, similar to DMSA. It has also been reported that *Lactobacillus* can inhibit brain damage caused by aging, trauma, Alzheimer's disease, and lipopolysaccharides (42–44). In particular, it has been demonstrated that *Lactobacillus fermentum* CQPC08 protects rats from Pb-induced oxidative damage by regulating the Keap1/Nrf2/ARE pathway (45). However, to the best of our knowledge, it has not been reported that *Lactobacillus casei* can alleviate Pb-induced brain damage. Furthermore, we also found that SYF-08 treatment was more effective in bringing about hippocampal damage recovery than the DMSA treatment. As Pb can compete with Ca^2+^ for nitric oxide synthase sites, influencing the responses to some neurotransmitters and interfering with neurotransmitter release and calcium levels in neurons, this finding might be explained by the binding of Ca^2+^ with DMSA.

The analysis of gut content from Pb-poisoned mice indicated that SYF-08 reduced intestinal damage, restored intestinal microflora dysbiosis, modulated bile acid metabolism, and downregulated the Pb-activated FXR-NLRP3 signaling pathway. It has also been elucidated that bile acid is crucial for Pb excretion. Additionally, medicines, such as thiamine and EDTA, and the probiotic, *Lactobacillus plantarum* LP33 attenuate Pb-induced injury by accelerating bile acid generation and Pb excretion (22, 46). Thus, this study is the first to report this relationship between *Lactobacillus casei* and bile acids in Pb poisoning models.

However, this study had some limitations. First, the types of bile acids that were influenced by SYF-08 during the Pb poisoning treatment were not identified. Second, the exact molecular mechanism by which SYF-08 activates FXR was not explored.

## Data Availability Statement

The datasets presented in this study can be found in online repositories. The names of the repository/repositories and accession number(s) can be found below: https://www.ncbi.nlm.nih.gov/, PRJNA818788.

## Ethics Statement

The animal study was reviewed and approved by the Institutional Animal Care and Use Committee of Southern Medical University (SMUL2018037).

## Author Contributions

Study design: HF, XM, and WZ. Data collection: ZC, ZT, JK, and LC. Data analysis: JL, YL, WH, WL, and JW. Manuscript preparation: ZC and ZT. All authors contributed to the article and approved the submitted version.

## Funding

This work was supported by National Natural Science Foundation of China (Nos. 31872630, 32070118, 81973071, and 81773473), Guangdong Basic and Applied Basic Research Foundation (No. 2019A1515011759), Guangdong Science and Technology Program key projects (Nos. 2018B020205002 and 2021B1212030014), and Medical Scientific Research Foundation of Guangdong Province of China (No. A2021191).

## Conflict of Interest

*Lactobacillus casei* SYF-08 belongs to Guangdong Huankai Microbial Science and Technology Co., Ltd. Any use of *Lactobacillus casei* SYF-08 without the permission of Guangdong Huankai Microbial Science and Technology Co., Ltd. is illegal. JW was employed by Guangdong Huankai Microbial Science and Technology Co., Ltd. The remaining authors declare that the research was conducted in the absence of any commercial or financial relationships that could be construed as a potential conflict of interest.

## Publisher's Note

All claims expressed in this article are solely those of the authors and do not necessarily represent those of their affiliated organizations, or those of the publisher, the editors and the reviewers. Any product that may be evaluated in this article, or claim that may be made by its manufacturer, is not guaranteed or endorsed by the publisher.
