# Supplementary material for: Lactobacillus casei SYF-08 Protects Against Pb-Induced Injury in Young Mice by Regulating Bile Acid Metabolism and Increasing Pb Excretion
Source: Front Nutr. 2022 Jun 28;9:914323. doi: 10.3389/fnut.2022.914323 (PMC9278719; doi:10.3389/fnut.2022.914323)
Supplement: Supplementary file 1 [file Data_Sheet_1.pdf]

Supplementary Material

Lactobacillus casei SYF-08 Protects against Pb-induced injury in young mice by regulating bile acid metabolism and increasing Pb excretion

Zhenhui Chen et al.

Supplementary Figure P2-7

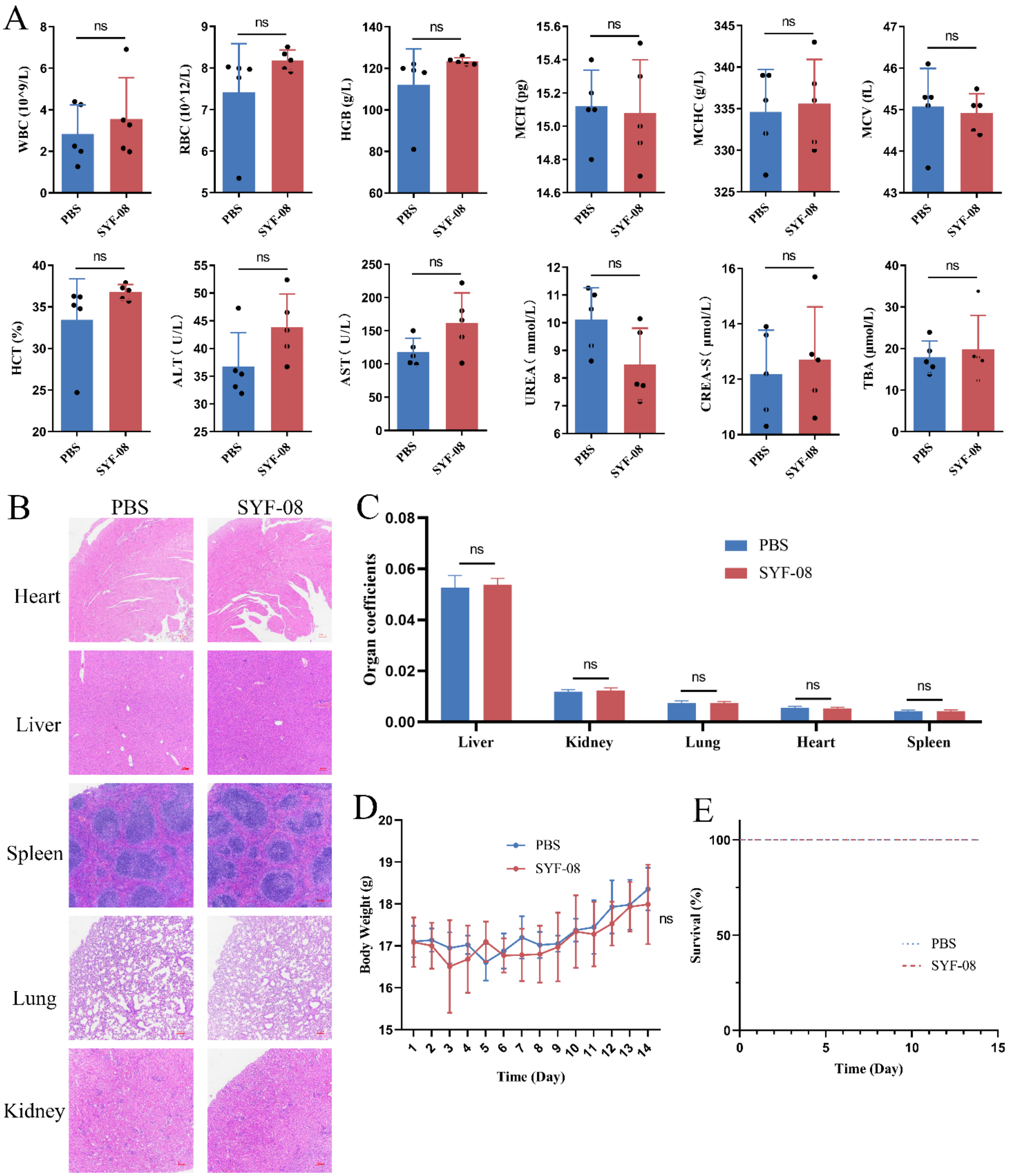

Supplementary Figure 1. Acute single dose oral toxicity test of SYF-08. (A) Hematological tests and serum biochemicals tests of the mice after the daily administration of PBS or SYF-08 for 7 days. The data show means + SD. P was calculated using two-tailed t-test. WBC white blood cells, RBC red blood cells, HGB hemoglobin, MCH mean corpuscular hemoglobin, MCHC mean corpuscular hemoglobinconcentration, MCV mean cell volume, HCT hematocrit, ALT alanine transferase, AST

aspartate transferase, UREA blood urea nitrogen, CREA-S creatinine, TBA total bile acid. (B) Represented HE images of the major organs of the mice after different treatments. Scale bar = 200 μm. (C) Organ coefficients of mice. P was calculated using two-tailed t-test. (D) Body weight of mice. P was calculated using two-tailed t-test. (E) Survival curves of mice. P was calculated using Log-rank (Mantel-Cox) test.

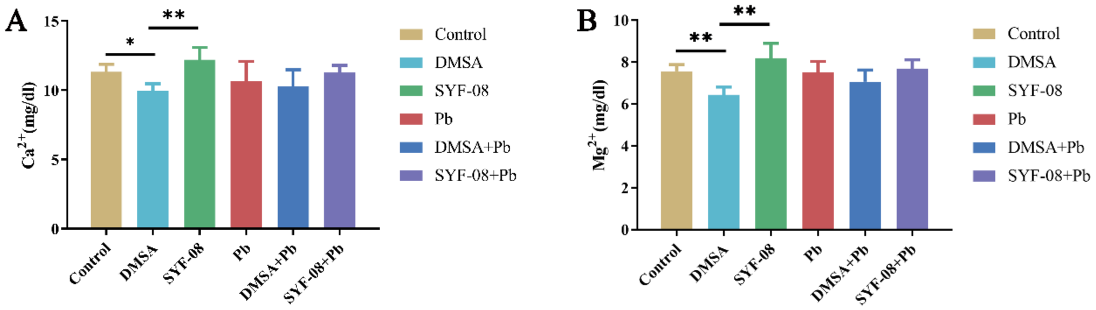

Supplementary Figure 2. The concentration of Ca^{2+} and Mg^{2+} in the blood. *P < 0.05; **P < 0.01.

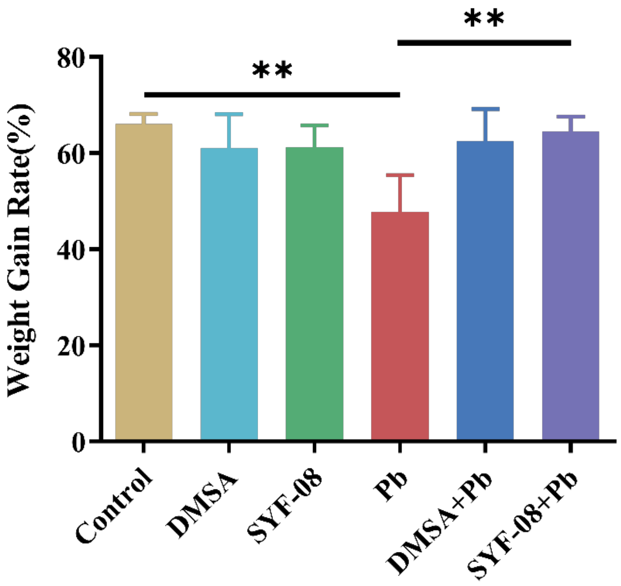

Supplementary Figure 3. The weight changed on the last day before euthanization. **P < 0.01.

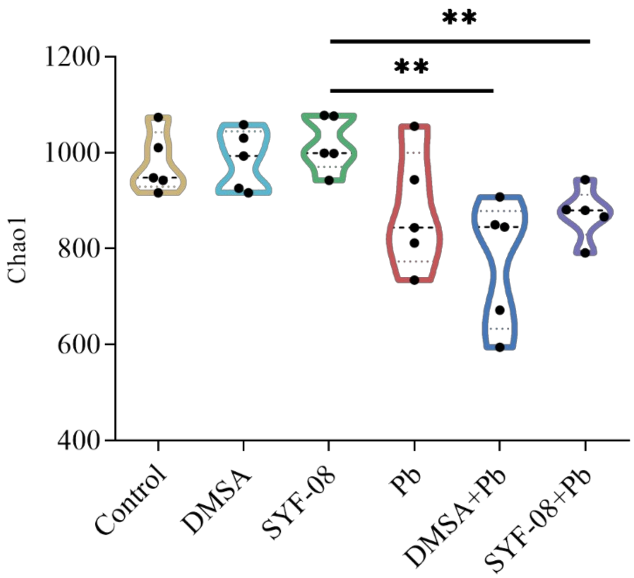

Supplementary Figure 4. The Chao1 indices in the different groups. **P < 0.01.

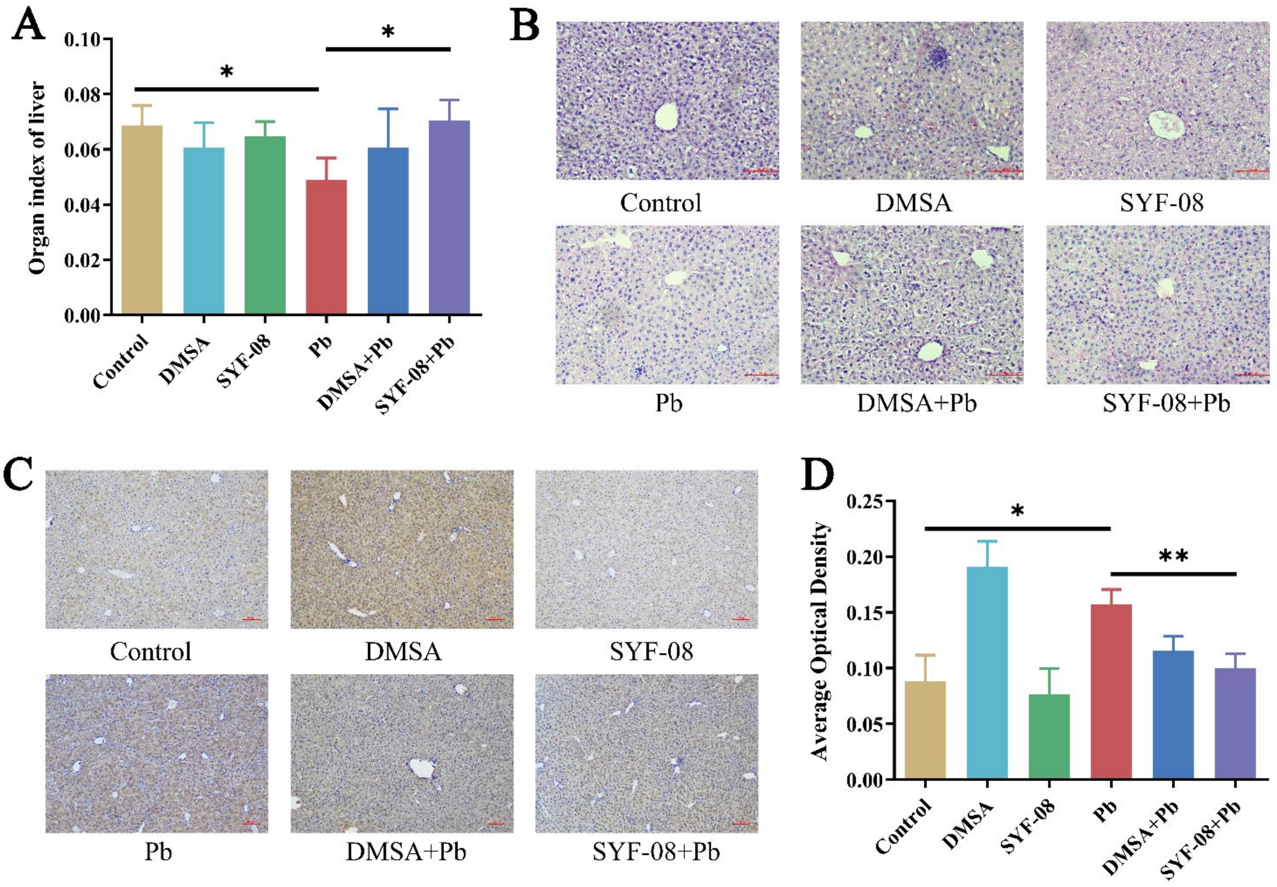

Supplementary Figure 5. *Lactobacillus casei* SYF-08 inhibits the activated FXR-NLRP3 signaling pathway in the liver. (A) The liver organ indices in the different groups. (B) Representative HE staining of liver in the different groups. (C) Representative IHC staining of FXR in liver. (D) The average optical density of FXR in different groups.*P < 0.05; **P < 0.01.
