# Supplementary figures and images for: Lactobacillus casei SYF-08 Protects Against Pb-Induced Injury in Young Mice by Regulating Bile Acid Metabolism and Increasing Pb Excretion
Source: Front Nutr. 2022 Jun 28;9:914323. doi: 10.3389/fnut.2022.914323 (PMC9278719; doi:10.3389/fnut.2022.914323)

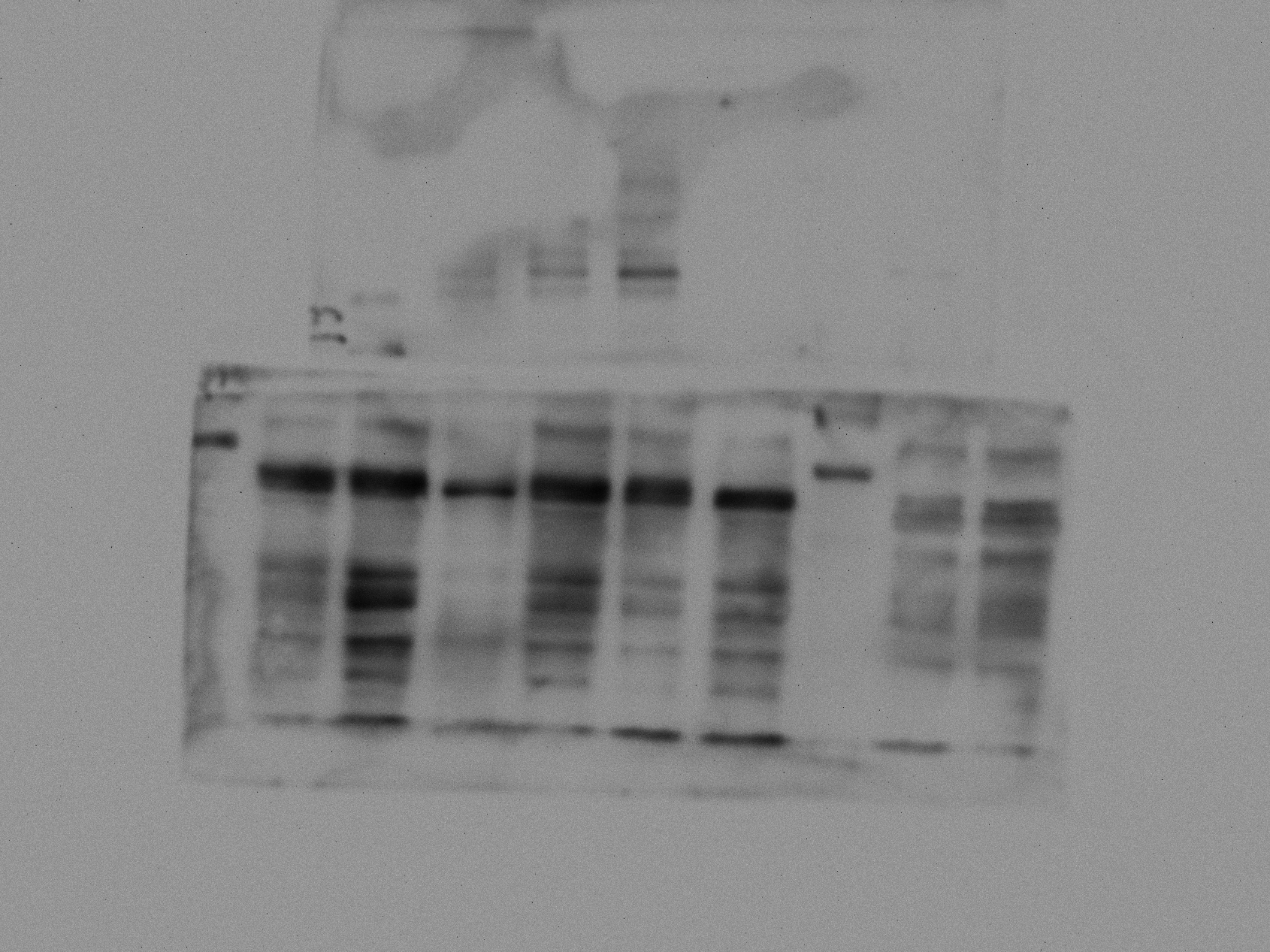

Supplement: Supplementary file 2 [file Data_Sheet_2.ZIP › Original Data/Figure.7/Caspase1.tif]

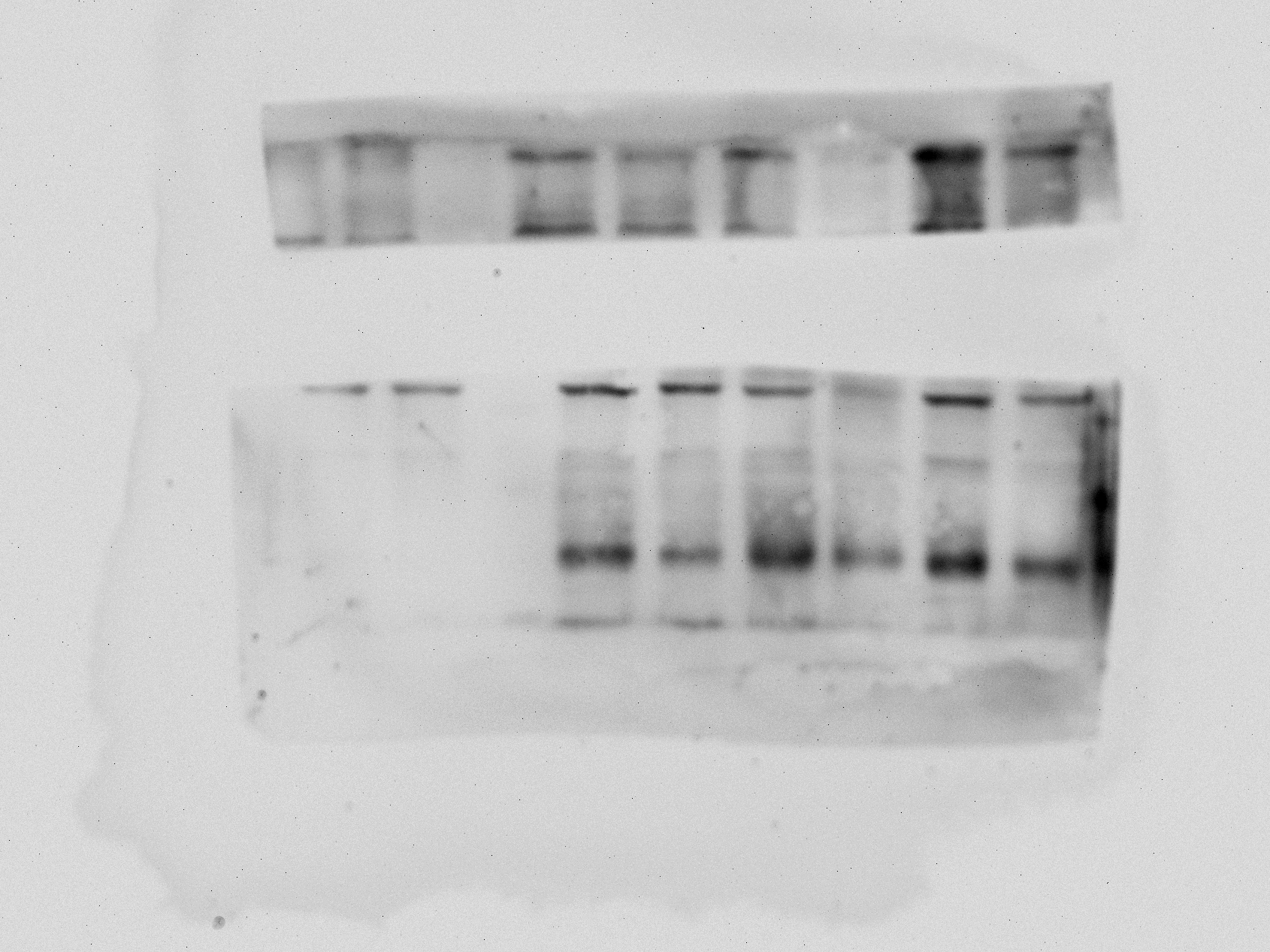

Supplement: Supplementary file 2 [file Data_Sheet_2.ZIP › Original Data/Figure.7/il-1β.tif]

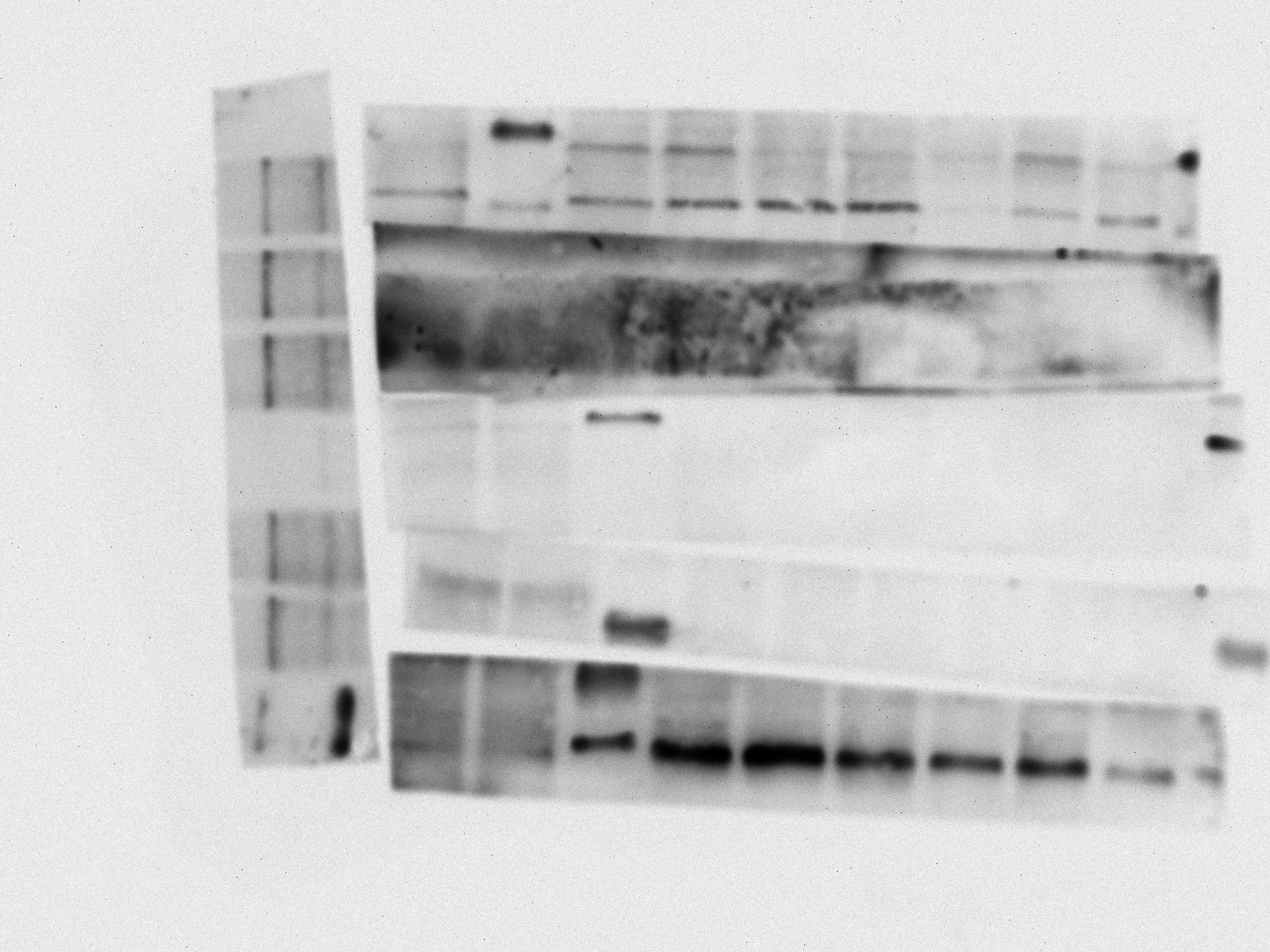

Supplement: Supplementary file 2 [file Data_Sheet_2.ZIP › Original Data/Figure.7/NLRP3.tif]

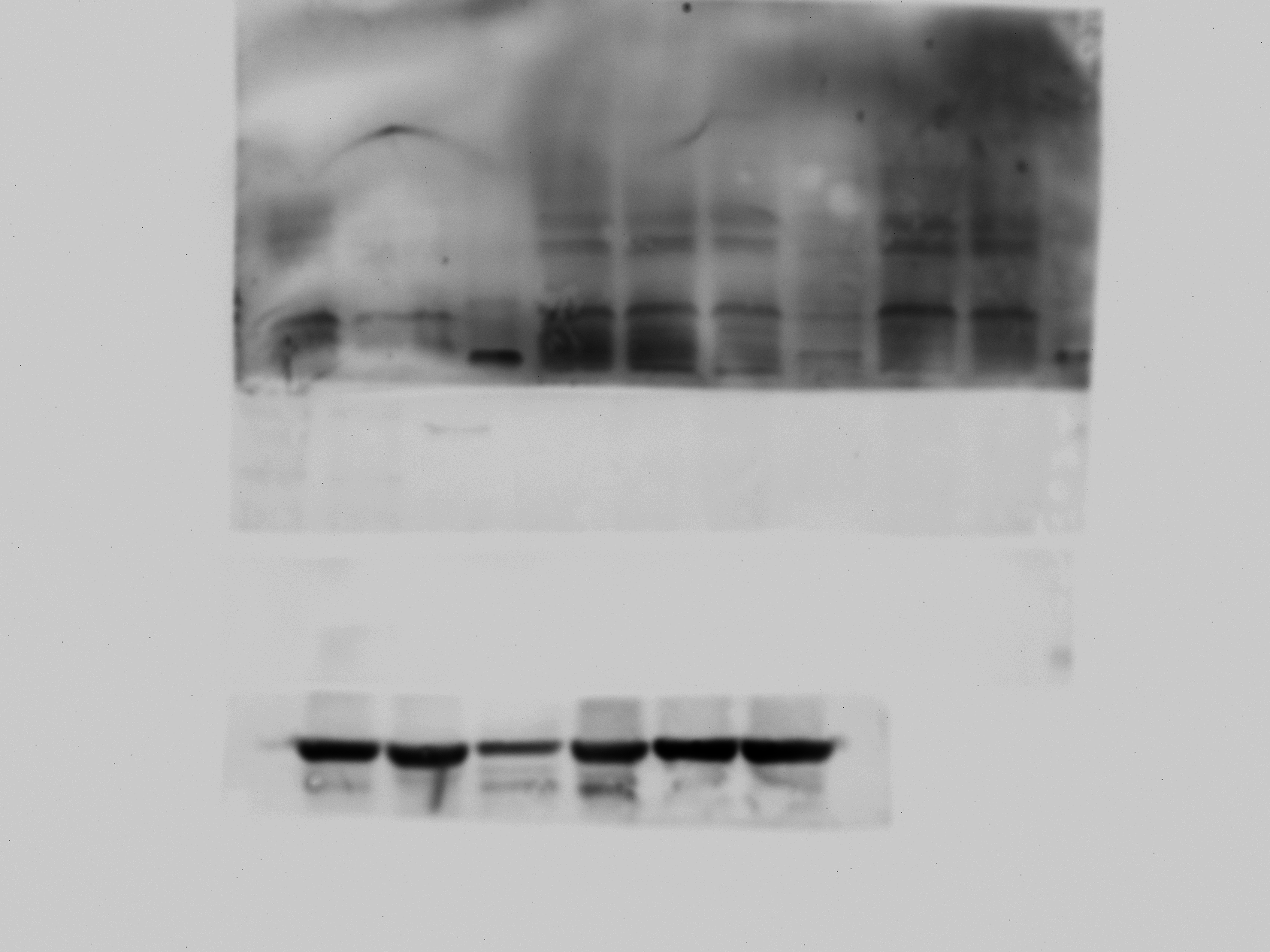

Supplement: Supplementary file 2 [file Data_Sheet_2.ZIP › Original Data/Figure.7/β-actin (G).tif]
